# Supplementary material for: Are carbon emissions trading and green financial instruments synergistic? -Comprehensive quantitative research based on content analysis
Source: PLoS One. 2024 Mar 7;19(3):e0298601. doi: 10.1371/journal.pone.0298601 (PMC10919601; doi:10.1371/journal.pone.0298601)
Supplement: S1 Data — (ZIP) [file pone.0298601.s001.zip › Data/地级市Z_F工作报告.zip!/║╙─╧/╔╠╟≡╩╨/└┤╫╘╣1⁄2╓┌║┼í╛┬φ┐╦╩2╛▌═°í┐_2015.docx]

各位代表：

　现在，我代表市人民政府，向大会报告工作，请予审议，并请各位政协委员和其他列席人员提出意见。

一、2014年工作回顾

2014年，在省委、省政府和市委的正确领导下，面对经济下行压力增大的形势，全市上下深入贯彻落实党的十八大、十八届三中、四中全会和习近平总书记系列重要讲话精神，抓住产业转移机遇，把握“持续求进、好中求快”的总基调，坚持以四项重点带全局，求真务实，锐意进取，较好完成了年初确定的目标任务。全市生产总值完成1697．6亿元，比上年增长9．2％；规模以上工业增加值完成383．4亿元，增长11．6％；固定资产投资完成1233．8亿元，增长19．5％；社会消费品零售总额完成579．7亿元，增长13．6％；一般公共预算收入完成100．7亿元，增长17．4％；城镇居民人均可支配收入达到22274元、农民人均纯收入8025元，分别增长10．2％和11．2％；全年居民消费价格上涨1．7％，控制在合理范围内。主要经济指标增速均高于全省平均水平，科学发展的基础更加坚实、支撑更加有力、前景更加广阔。

（一）产业结构不断优化。把工业作为稳增长、调结构的主阵地，稳步提升二三产业在生产总值中的比重，全市产业结构为22．4∶46．1∶31．5。认真落实国家宏观调控政策，强化重点区域、重点行业、重点企业运行调节，加大对中小企业扶持力度，搭建产销对接平台，成功举办了电动车、纺织服装、食品博览会。做好要素保障，加大信贷投入，抓好土地管理、环境综合整治，有效缓解了资金、土地、环境容量等瓶颈制约。把产业集聚区作为工业发展的主要载体，按照“四集一转”要求，突出主导产业集群发展，重点推进基础设施、科技创新、公共服务，产业集聚区建设迈上新台阶。全市产业集聚区完成固定资产投资1081．2亿元，增长31．7％，高于全市投资增速12个百分点，占全市投资的87．6％；规模以上工业企业达到528家，完成增加值286．5亿元，增长21．6％，占全市规模以上工业增加值的74．8％。民权制冷、柘城金刚石、睢县制鞋、夏邑纺织服装、虞城五金电子、宁陵农资化工等主导产业特色突出；睢阳区针织服装、梁园区生物医药、开发区电动车和环保装备产业，都形成了比较强的生产能力，具备了区域竞争优势。加快培育新材料、生物医药、环保装备等新兴产业和高新技术产业，全市高新技术产业增加值占规模以上工业增加值的比重提高到25．8％，六大高成长产业完成工业增加值217．6亿元，增长14．1％，对工业增长贡献率达到66．7％。加强检验检测公共技术服务平台建设，建成国家级质检中心2家、省级质检中心4家。大力发展现代服务业，把商务中心区和特色商业区作为发展服务业的重要载体，引进总部经济，突出发展现代物流、电子商务、金融保险、文化旅游等现代服务业，国药物流、华润医药物流、多美滋物流配送中心等一批重点项目在我市落地，中铁快运、南航快运、中通、申通等国内知名物流企业在我市设立分支机构。全市商品交易市场快速发展，年交易额10亿元以上的市场12家，农产品中心批发市场、中原车城销售连创新高，交易额达到228亿元、126亿元。金融业快速发展，年末人民币各项存款余额1759．9亿元，增长12．9％；贷款余额1007．2亿元，增长20．9％。金融机构引进实现历史性突破，浦发银行、郑州银行成功落户商丘，睢县、宁陵县村镇银行相继开业。软件开发、呼叫中心、信息咨询、服务外包等三方服务业发展迅速，电子商务、网上购物等新兴业态势头强劲。阿里巴巴�商丘产业带入驻企业617家，产业带客流量和转化率位居全国前列。文化旅游业加快发展，7月5日迎来了首趟从上海到商丘的旅游专列，全年接待游客962．9万人次，旅游收入19．6亿元。

（二）现代农业稳步发展。全年粮食总产达到107亿斤，实现“十一连增”，连续三年被评为全国粮食生产先进市。畜牧业平稳增长，全年肉类、禽蛋、奶类总产量分别为48．1万吨、23．2万吨、28．6万吨。农业产业化快速发展，市级以上龙头企业252家，销售收入超亿元的88家，科迪乳业、诚实人面业、宏伟饮品等8个产业化集群被认定为省农业产业化集群。开展农田水利基础设施、水库除险加固、引黄调蓄工程建设，新建高标准粮田89万亩，改善有效灌溉面积53万亩、除涝面积65万亩、引黄灌区配套面积21万亩，新增旱涝保收田17万亩。农机总动力1012万千瓦，农作物综合机械化水平达到83．5％。完成造林10．7万亩，国家森林城市创建规划获得国家林业局批准。

（三）新型城镇化加速推进。统筹发展中心城区、县城、重点镇和新农村，四级城乡规划建设体系进一步完善。全市城镇人口达到340．5万人，城镇化率达到45．2％，中心城区人口达到120万人。启动了城市总体规划（2005～2020）修编，完成了梁园特色商业区、火车站核心区等11个片区控制性详细规划编制，市区控规覆盖率达到90％以上。加快郑徐高铁商丘站区、商务中心区、古城保护区三大区域建设。实施神火大道、睢阳大道、中州路等道路改造升级工程，积极推进华夏游乐园、汉梁文化公园建设。开展康林河、忠民河等6条河道治理，启动中心城区3个污水处理厂建设；实施河流清洁碧水行动，促进了大沙河、包河、运河等河道水质改善。开展农村土地综合整治，严厉打击违法用地，规范土地出让行为，保障了发展用地需求。继续推进“六城”联创，开展了小广告、门头牌匾、违法建设等十个专项整治行动，依法拆除各类违法违章建筑12000多处、40多万平方米，市容市貌明显改观。围绕商务中心区和特色商业区建设，着力提高县城综合承载能力，县城功能不断完善，形象得到提升。深入开展“美丽商丘�整洁村镇”活动，我市被列为全省改善农村人居环境试点。

（四）招商引资和项目建设力度加大。围绕主导产业，持续开展大招商活动，成功举办了2014中国�商丘国际华商节，引进了一大批知名企业和项目。全市实际利用省外资金496．1亿元，利用外资3．1亿美元，外贸进出口总额达到3．4亿美元，增速为35％。狠抓中心城区招商，王老吉凉茶、舒华体育等项目落地，天骏电动汽车、快菱电梯、传化公路港等项目达成合作意向。深入开展“项目服务年”活动，以项目建设带动投资快速增长。市管重点项目完成投资905亿元，增长28％。富士康实训工厂、顿汉格力、史丹利、英泰冷藏车等80个项目竣工投产。郑徐客专商丘段、连霍高速拓宽改造、商登高速、郑民高速二期建设按计划推进。商合杭客专商丘段可行性研究报告已由国家发改委组织评审；沱浍河航运夏邑段施工图已通过省交通厅评审；商丘机场可研报告的前置性文件全部得到国家有关部委批复，项目设计已委托空军设计研究局编制。

（五）改革创新进一步深化。圆满完成市级政府机构改革，市政府工作部门由34个减少到32个，有序推进工商、质监、食品药品监管等部门职责和机构整合，深入推进事业单位分类、医药卫生体制改革。理顺了城乡一体化示范区管理体制。深入推进国有企业改革，稳步推进商电铝业集团依法破产，积极帮助河南路桥集团脱困发展。深化行政审批制度改革，取消、下放行政审批项目45项。积极推进工商登记制度改革，简化手续，降低门槛，放宽企业注册登记条件，各类市场主体达到20．7万户，增长18．1％。扎实推进财税改革，完善全面规范、公开透明的预算制度，从严控制一般性支出，确保了“三公”经费只减不增。推动关键领域科技创新，新认定国家级高新技术产业化基地1家、国家级科技企业孵化器1家，争取省级重大科技专项2项，培育发展高新技术企业12家，发展省级工程技术研究中心6家，高新技术产业增加值完成98．8亿元，增长37．6％，高于规模以上工业增加值增速26个百分点。

（六）各项社会事业协调发展。加大民生投入，全市财政民生支出284．8亿元，增长14．2％，占一般公共预算支出的80．8％，比上年提高1．5个百分点。全市城镇新增就业7．8万人，下岗失业人员再就业2．9万人，农村劳动力转移就业7．2万人。文化、教育、体育、医疗卫生等基本公共服务体系不断完善，城区办学条件有所改善，商丘幼师新校区投入使用。大运河通济渠商丘南关段和夏邑济阳段申遗成功，被列入世界文化遗产。“舞台艺术送农民”、“百戏进百乡”、“农村公益电影放映工程”完成了年度计划。城乡居民养老、医疗、工伤和失业保险覆盖率都达到95％以上。新农合全市参合农民617．2万人，参合率99．3％。全年人口自然增长率4．99‰，政策生育率98％。开工各类保障房项目65个、82854套。发放住房公积金贷款18．5亿元，解决1．5万户困难职工住房问题。加快农村安全饮水工程建设，解决了104．4万农村居民和学校师生的饮水安全问题。高度重视生态环境建设，实施蓝天、碧水和乡村清洁工程，积极淘汰造纸、电解铝、制革、酒精等落后产能，提前一年完成省“十二五”淘汰落后产能任务，环保责任目标年度考核位居全省第三位。加强民族宗教工作，促进民族团结进步、宗教关系和谐稳定。重视信访工作，及时排查解决人民群众反映的问题。加强城乡社区建设，完成第八届村委会换届选举。加强社会治安综合治理，严厉打击非法集资和刑事犯罪，加大反暴力反恐怖、打击黑恶势力、扫除“黄赌毒”力度，平安商丘建设取得新成效，群众安全感、满意度不断提升，被评为全省平安建设工作先进市。抓好安全生产和食品药品安全工作，全市安全生产形势持续平稳。

（七）民主法治和反腐倡廉建设不断加强。自觉接受人大依法监督、政协民主监督和社会各界监督，办理人大代表建议和政协委员提案699件，满意率达到98％。深入开展党的群众路线教育实践活动，认真执行中央八项规定，重点解决“四风”突出问题，政府行政效能、公务员能力素质得到提升。全面推进依法治市，落实“六五”普法规划，认真开展法制宣传教育。加强行政监察、行政复议、行政执法监督和审计监督，强化廉洁从政，反腐倡廉取得新成效。加强社会主义核心价值观教育和精神文明建设，“商丘好人”群体不断扩大，“一言九鼎，实干兴商”城市精神，日益成为广大干部群众的自觉行动。

开展“双拥”共建活动，推动军民融合深度发展，驻商解放军、武警部队和民兵预备役在经济社会发展中作出了积极贡献，巩固了军政军民团结的良好局面。工会、共青团、妇联等人民团体在经济社会发展中发挥了不可替代的作用。外事侨务、对台、统计、气象、地震、人防、档案、史志等工作取得了新的成绩。

各位代表！

　这些成绩的取得，是省委、省政府和市委正确领导的结果，是市人大、市政协监督支持的结果，是全市人民共同奋斗的结果。在此，我代表市人民政府，向广大工人、农民、知识分子、干部，向人民解放军指战员、武警消防官兵、公安民警，向关心、支持、参与商丘建设的社会各界人士，表示衷心的感谢！

　在肯定成绩的同时，我们也清醒地认识到，经济社会发展和政府自身建设还存在一些突出问题：一是产业结构不优，工业不够强，服务业水平比较低，消费需求不足，煤电铝等行业、企业经营困难，效益下滑，农业持续增产、农民稳定增收难度增大，一些领域体制机制障碍仍然较多；二是城镇化进程中产业支撑能力不足，城乡二元结构比较突出，城镇基础设施不完善，中心城区辐射带动能力不强，城市管理水平不高；三是资金、土地、用工等生产要素制约依然存在，资源环境约束加剧；四是民生保障能力不强，一些涉及群众切身利益的问题还没有得到解决，改善民生、维护社会稳定的任务依然繁重；五是政府职能转变、简政放权还不到位，部分工作人员责任意识淡漠、履职能力不强、工作作风不实，行政服务效能还需要提升，反腐倡廉工作需要进一步加强。对此，我们一定高度重视，在今后的工作中认真解决。

二、2015年工作总体要求和主要目标

今年是全面深化改革的关键之年，是全面推进依法治国的开局之年，是全面完成“十二五”规划的收官之年，也是《河南省全面建成小康社会加快现代化建设战略纲要》的启动之年。今年政府工作的总体要求是：以党的十八大和十八届三中、四中全会精神为指导，认真贯彻落实省委和市委决策部署，坚持“持续求进、好中求快”的总基调，以建设承接产业转移示范市为统揽，以三区发展促突破，以四项重点带全局，以五大建设作保障，稳增长，调结构，促改革，扩开放，控风险，惠民生，把握新常态，抓住新机遇，增创新优势，实现新发展，不断开创我市经济社会持续健康发展新局面。

今年经济社会发展主要预期目标是：在优化结构、提高质量、增加效益的基础上，全市生产总值增长8．5％；一般公共预算收入增长10％；全社会固定资产投资增长17％；社会消费品零售总额增长13％；进出口总额增长10％；实际利用外资增长8％；人口自然增长率控制在7．1‰以内；城乡居民收入与经济增长同步，居民消费价格涨幅控制在3％左右；节能减排和环境质量改善完成省定目标；发展方式转变取得新进展。

实现上述要求和目标，必须准确把握经济发展新常态的大趋势、大背景，立足商丘，找准定位，明确目标，把发展重点转到提高质量和效益上来，坚持把握好六个方面的着力点。一是坚持加快发展。商丘是传统农业市，经济总量比较小，工业化、城镇化水平比较低，第一要务是发展。必须毫不动摇地坚持以经济建设为中心，一心一意谋发展，在加快发展中实现转型升级，提高发展质量。二是坚持深化改革。把改革作为破解瓶颈制约的根本之策，通过政府职能转变、体制机制创新、优化审批流程和深化土地、户籍、国有企业等领域的改革，努力解决阻碍经济发展的困难和问题，增强经济发展活力。三是坚持优化结构。针对经济发展中不全面、不协调、不可持续的问题，以市场为导向，结合国家淘汰落后产能和化解过剩产能的政策，调整优化产业结构和产品结构，拓展新型城镇化、新型工业化、农业现代化和信息化发展空间。四是坚持培育优势。适应新常态，必须立足商丘，扬长避短，主动作为，建设大交通、发展大物流、培育大产业，增强竞争力。五是坚持开放引领。把扩大开放作为应对复杂局面、促进改革发展最有效的举措，坚定不移实施开放带动战略，大力发展外向型经济，积极引进行业龙头企业和产业链上的配套企业，壮大产业集群。六是坚持创新驱动。深化科技创新体制改革，强化企业创新主体地位，建设研发平台，培育技术创新体系，不断提高创新水平。

三、2015年重点工作

　今年工作任务非常繁重，我们将重点做好八个方面的工作。

　（一）加快构建现代产业体系。一是强化工业主导地位。持续推动产业结构优化升级，促进工业经济向高端、高质、高效方向发展。改造提升传统产业，重点围绕冶金建材、制冷装备、纺织服装、电动车、食品加工等传统产业，引导企业升级改造，向产业价值高端发展，提高市场竞争力。培育发展战略性新兴产业，重点围绕电子信息、生物医药、新材料、环保装备等优势产业，积极承接产业转移，促进企业集中集聚，形成一批先导性、带动性强的新兴产业。加强工业经济运行调节，综合运用延伸链条、技术改造、兼并重组、淘汰落后产能等手段，缓解资金、土地、用工等要素制约，提升产业综合竞争能力。落实支持中小微企业、民营经济发展的各项政策，激发市场活力和企业创造力。推进工业化和信息化深度融合，实施生产设备的数字化、智能化、网络化改造，普及生产管理智能化和营销服务网络化，推动信息化与生产制造、技术创新、现代服务、节能环保等方面融合发展。继续举办针织童装、电动车、食品博览会，举办“中国�民权首届制冷装备博览会”，深化产销对接，帮助企业开拓市场。二是大力发展服务业。把商务中心区和特色商业区作为现代服务业发展的重要平台，采取有力措施，加快建设区域性服务业中心。突出发展现代物流业，加快豫东综合物流集聚区建设，完善基础设施功能，引进品牌物流企业，积极申报综合保税区。加快商品交易平台建设，运用现代技术管理手段对传统商贸物流企业进行改造提升，完善现代市场体系。围绕火车站区域改造和高铁商城建设，打造市区北部高档商业圈。积极发展文化产业，把商丘古城、睢县北湖、民权王公庄、夏邑火店镇等文化产业园打造成我市示范产业园区，培育知名文化品牌和重点文化企业。做好旅游宣传与推介，积极发展文化体验、生态休闲、果蔬采摘等旅游业态，加大A级景区、星级饭店、星级旅行社创建力度，力争新增2处以上国家A级景区。发展金融服务业，争取交通银行、中信银行进驻商丘，新增1～2家村镇银行，争取引进5家以上保险机构。继续做好科迪乳业、东和环保等企业上市工作，争取1家企业上市、2家以上企业在新三板挂牌。做好预防和处置非法集资工作，加强对信用类理财公司的监管，加大非法集资打击力度，有效防范金融风险。发展新兴服务业，加快发展科技研发、信息咨询、检验检测等生产性服务业，积极发展楼宇经济、健康养老、教育培训等生活性服务业。以电子商务为牵引，支持建设电商产业园，继续推进与阿里巴巴的战略合作，壮大阿里巴巴�商丘产业带，力争年底入驻企业突破1000家。三是打造产业发展平台。把产业集聚区作为推进产业发展的重要载体，坚持“四集一转”，完善基础配套，壮大产业集群，促进提速提质。围绕主导产业，积极引进龙头企业和配套企业，努力培育规模效益明显、产业链条完整、配套体系完善、竞争优势明显的产业集群。争取装备制造产业集群主营业务收入超过500亿元，纺织服装产业集群超过700亿元，食品加工产业集群超过700亿元。加快产业集聚区道路、电力、通信、污水处理等基础设施建设，推动综合服务、检验检测、信息咨询、技术研发、物流配送等公共服务平台建设，提升综合承载能力。四是强化创新驱动发展。组织实施科技创新主体培育工程，提升现有科技孵化器的功能，培育一批高新技术企业、科技型中小企业，新增高新技术企业5家以上，积极创建河南省商虞高新技术产业开发区。围绕产业集聚区主导产业发展，加快省级研发中心、院士工作站、产业技术创新战略联盟建设，突破一批核心技术，建立基本技术标准，培植自主品牌，高新技术产业增加值超过116亿元，增长18％以上，占规模以上工业增加值的比重达到28％。

（二）做强做优现代农业。一是稳定提高粮食生产能力。按照“稳粮增收、提质增效、创新驱动”的总要求，实施高标准粮田百千万工程，建成高标准粮田75万亩。加快中低产田改造，推广良种良法配套、农机农艺融合等农业科技，确保全市粮食综合生产能力稳定在100亿斤以上。二是培育现代农业产业化集群。抓住国家支持商丘建设现代农业示范区的重大机遇，积极推进现代农业产业园区、农产品集中区和农产品市场体系“三大载体”建设，采取项目引导、政策倾斜等措施，壮大龙头企业实力，延伸产业链条，提升产业层次，增加农产品附加值，打造一批“全链条、全循环、高质量、高效益”的农业产业化集群。新发展市级农业产业化重点龙头企业10个，形成销售收入10亿元以上的产业化集群8个、20亿元以上的2个。三是调整农业结构。按照“一乡一业”、“一村一品”的要求，大力发展果蔬花卉种植等高效农业、生态农业、创意农业、休闲农业，建设现代农业生产基地、示范园区和都市农业工程。大力发展畜牧业，推动奶牛、生猪、家禽、肉牛肉羊集群发展，加快建设标准化、规模化的生产基地。推进食品农产品出口示范区建设，扩大出口规模，带动农民增收。加强农产品质量监测体系建设，确保农产品质量安全。四是加强农业基础设施建设。搞好农村道路、电力、安全饮水、通信等基础设施建设。实施农业综合开发、灌区续建配套与节水改造工程，完善农田水利配套设施，提高抗旱排涝标准，新增和改善灌溉面积72万亩、除涝面积77万亩，新增旱涝保收田20万亩。完善气象防灾减灾机制，推进气象现代化建设，提高防御灾害能力。五是做好土地流转工作。加快农村土地承包经营权确权颁证，健全土地流转机制，建立规范有序的土地流转平台，强化信息沟通、政策咨询、合同签订、价格评估等服务，按照依法、自愿、有偿的原则，引导和鼓励农民采取转包、出租、互换、入股等形式，将土地向种养大户、家庭农场、农民合作社、龙头企业流转，发展农业适度规模经营。六是搞好扶贫开发。巩固发展专项扶贫、行业扶贫、社会扶贫“三位一体”扶贫格局，千方百计增加农民收入，不断提高贫困地区和贫困人口的自我发展能力。继续实施好我市大别山区域扶贫攻坚与小康建设规划，做到村户精准扶贫，实现13万贫困人口摆脱贫困。

（三）科学推进新型城镇化。按照“一城、六组团、24个重点镇、社会主义新农村”四位一体的总体布局，加快构建新型城镇化体系，在完善功能、统筹发展上下功夫。一是强力推进中心城区建设。发挥规划引领调控作用，完成《商丘市城市总体规划（2015～2030）》编制，强化规划管理，维护规划的严肃性和权威性。以郑徐高铁商丘站区、古城保护区、商务中心区、开发区、物流园区、梁园产业聚集区、睢阳产业聚集区、食品工业园区等八大板块建设为重点，强力推进道路管网、园林绿化、内河水系等城市基础设施建设。发挥示范区的示范带动作用，坚持产业、工农、城乡统筹发展，逐步把示范区建成工业、服务业和都市生态农业协调发展的复合型经济区。加快公共服务设施建设，促进教育、文化、医疗、体育等高端要素向中心城区集中，提高中心城区辐射带动能力。提升城市管理水平，按照“分级管理、权责统一、重心下移、以区为主”的工作思路，深化城市管理体制改革，积极推行网格化管理。推进数字化城市建设，实现市区两级平台和终端平台的网络对接。继续开展“六城”联创活动，深入推进省级文明城、全国卫生城创建工作，完成国家森林城市创建任务，争创全国双拥模范城七连冠，巩固国家旅游城、园林城创建成果。二是大力发展县城组团。按照“改造老城、发展新区”的工作思路，加快县城新城区、产业集聚区、商务中心区和特色商业区等功能区开发，推进旧城改造步伐，引进更多的工业项目在产业聚集区布局，更多的服务业项目在商务中心区和特色商业区集聚，增强县城辐射带动能力。三是有序推进小城镇建设。加快建设24个区位优势好、产业基础强的小城镇，结合各自资源禀赋，引导各种要素向重点镇布局，推动交通、通信、文化、卫生、物流快递向重点镇延伸，加快形成服务周边的生产生活中心，增强吸纳农村人口转移能力。四是稳步推进新农村建设。坚持“五规合一”，分类推进新农村综合发展、特色发展。认真做好新农村规划编制，调整优化村庄布局，合理确定村庄规模，引导村民按照规划进行建设。加强农村人居环境整治，持续推进“美丽商丘�整洁村镇”活动，探索建立垃圾收集清运、污水处理等环境卫生管护长效机制，使更多村庄成为宜居的美丽家园。

（四）持续深化改革。按照市委全面深化改革意见，扎实推进我市第一批改革事项。一是深化行政体制改革。巩固政府机构改革成果，制定“三定”工作方案，全面完成市县政府机构改革。围绕简政放权，继续取消、下放行政审批事项，全部取消非行政许可审批，清理和规范中介服务事项。借鉴“五险合一”经验，实现社会保险经办管理“一站式”服务，建立便民快捷的社会保险经办管理服务体系。完成党政机关公务用车制度改革。二是深化国有企业改革。围绕激发市场活力，通过兼并重组，推进国有企业产权多元化，积极稳妥发展混合所有制经济，鼓励民间投资主体参与国企改制重组，实现投资主体和产权多元化，依法做好商电铝业破产工作。加快国有经营性文化单位转企改制，支持文化企业发展壮大。三是深化财税体制改革。认真落实新预算法，加强政府性债务管理，建立跨年度预算平衡机制，推进政府和部门预决算公开，确保“三公”经费只减不增。四是深化价格改革。积极稳妥推进水、电、油、气等资源性产品价格改革，完善阶梯电价、水价政策，探索排污费征收调整机制，实行差别化排污收费政策。五是深化农村改革。抓好农村产权制度改革，加快农村土地承包经营权、农村集体建设用地确权登记颁证工作，谋划建设农村产权交易市场，建立健全市、县、乡、村四级土地流转服务体系。六是加快户籍制度改革和医药卫生体制改革。深入落实户籍制度改革措施，推进教育、医疗、就业、社保等配套改革，推进农业转移人口市民化。全面深化县级公立医院综合改革，完善全民医保体系，实施城乡居民大病保险，深入推进社会办医。

　（五）强力实施开放招商。把招商引资作为“以不变应万变”的有效举措，抢抓机遇，大力承接产业转移。一是明确招商重点。突出重点区域，主攻东部沿海发达城市群体，建立珠三角、长三角及港澳地区招商战区。突出主导产业，重点引进制冷、食品、纺织服装、新材料、生物医药等产业，增强竞争力。突出招大引强，瞄准国内外500强企业、著名品牌、上市公司、行业龙头，重点引进跨国集团、央企、省企、大型民企以及配套服务的中小型企业。突出链条招商，围绕主导产业开展“延链、补链、强链”招商，争取产业集群整体引进，形成带动效应。二是做强招商平台。抓好产业集聚区、商务中心区和特色商业区建设，培育产业集群，尽快形成一批具有较强竞争力的千亿元主导产业集群和百亿元特色产业集群，形成一批具有鲜明特色的服务业集群。2015年，全市产业集聚区新落地亿元以上主导产业项目100个，建成投产项目100个，完成工业项目投资850亿元。三是提升服务水平。不断创新服务机制，优化发展环境，继续实行领导分包项目、服务承诺、限时办结等工作机制，重点推行代办、代建、代招、代训等工作机制。对确定的重点项目，明确一名领导、指定一个部门、组建一套班子，提供全程服务。开展专项整治，打击影响企业发展的各类不法行为，营造宽松的发展环境。四是抓好项目推介。积极组织企业和项目“走出去”，充分利用招商平台，做好项目包装和推介，寻求新的合作伙伴和合作方式，在引进境外资金上实现新突破。在对外直接投资、承包工程、劳务合作上下功夫，带动设备出口和外派劳务。五是加强中心城区招商。立足中心城区产业发展基础，围绕纺织服装、装备制造、食品、医药等主导产业，按规划布局，发挥中心城区优势，吸引更多项目落户中心城区，打造雁阵效应。引导金融、研发、总部、物流、中介等服务业项目向商务中心区、城乡一体化示范区集聚，提升辐射带动能力。

　（六）加强支撑能力建设。以重点项目实施为载体，推进基础能力建设，为经济社会发展奠定坚实基础。一是打造综合交通体系。以建设中原经济区综合交通枢纽为目标，加快实施郑徐客专、连霍高速拓宽改造项目，确保按时完工；推进商合杭客专、沱浍河航运夏邑段、商丘机场等重大项目前期工作，争取早日开工建设；营造良好施工环境，确保商登高速、郑民高速如期建成通车，在商丘境内形成“三纵三横”高速公路网，实现“县县通高速”目标。积极推进干线公路升级改造，加快实施农村公路“三年行动计划”，提升道路畅通能力。二是提升信息化应用水平。以国家智慧城市、数字城市建设为契机，加快城乡信息资源管理和服务平台建设。积极发展信息服务业，今年全市信息消费规模增长25％以上，打造2个市级、1个省级“两化”融合试验区。三是加强能源基础支撑。积极推进与中电国际合作，争取商丘市2�35万千瓦热电联产项目年内开工建设。加快电网升级改造和西气东输豫东支线二期工程，积极发展太阳能光伏发电、垃圾发电，扩大生物柴油等新能源产能。四是抓好重点水利项目建设。推进郑阁、王安庄、任庄、马楼等水库除险加固工程，完成虬龙沟、小堤河、沱河、黄河故道等中小河流治理工程，构建复合型、多功能的水利网络。五是加强人才支撑。大力发展职业教育，围绕产业集聚区主导产业和骨干企业用工需求，灵活设置相关专业，开展订单式培训。加强校企合作，鼓励学校在企业设立实训基地。创新人才培养选拔、评价使用、流动配置、激励保障等机制，引导科技人才、工程技术人才向产业一线集聚。六是加强项目基础支撑。围绕基础设施和主导产业，千方百计做好项目引进和建设工作。今年全市第一批计划安排省、市重点建设项目210个，总投资2208亿元；今年计划投资900亿元，增长20％。创新项目管理机制，确保万宝家电年产120万台冰箱冰柜、赛琪年产200万件服装、200万件鞋包，金锐金刚石产业园、中原佳海国际商贸城、古城棚户区改造安置等重大工业、商贸物流、城市建设和民生项目顺利推进。扎实做好“十三五”规划编制工作，加强项目谋划，计划入库项目1000个以上，总投资4000亿元左右，“十三五”期间完成投资3500亿元，争取一批重大项目进入全省、全国的盘子，为我市今后发展奠定基础。

　（七）加强生态文明建设。把生态文明建设融入经济社会发展全过程，坚持节约集约，推动绿色发展，增强可持续发展能力。一是大力发展循环经济。提升循环经济发展水平，以产业集聚区和重点企业为依托，重点打造生态农业、环保产业以及建筑领域循环产业链，加快形成节约资源和保护环境的发展模式。提高准入门槛，实行项目审批与区域环境质量、产业结构调整、节能减排绩效挂钩。二是加大治污减排力度。强化减排精细化管理，狠抓重点减排项目建设和运行监管，把结构减排、工程减排、管理减排和科技减排的各项措施落实到位。淘汰、改造高排放项目，实行挂牌督办，坚决关停一批污染严重、治理无望的企业，严格控制区域排污总量。开展节能行动，实施节能技术改造，完成企业转型升级节能专案。三是强化环境综合治理。实施“蓝天工程”，制定重污染天气应急预案，积极推进电力机组脱硫脱硝、城市燃煤锅炉改造，严格控制城市施工和道路扬尘，开展餐饮业油烟污染治理；实施“碧水工程”，开展重点河流环境综合整治，确保饮用水水源地水质达标率100％；实施“乡村清洁工程”，以生活污水和生活垃圾治理为重点，开展农村环境综合整治，努力营造天蓝地绿水净宜居环境。四是严格环境执法。加强新《环保法》的宣传和贯彻落实。开展重点污染源执法监管活动，加强对重点污染源、重点建设项目、重点流域检查，坚决查处违法建设、违法生产、无证排污、违法排污、超标排污等行为。深化污染综合防控，强化辐射环境管理，推动清洁生产。五是抓好林业生态建设。以创建国家森林城市为抓手，围绕40项创建指标，大力实施生态廊道、农田防护林、平原沙荒造林、特色经济林、花卉苗木等林业生态建设提升工程，全年造林15．6万亩，森林抚育和改造5万亩，达到国家森林城市标准。

　（八）切实保障改善民生。一是确保就业稳定增长。实施全民技能振兴工程，同步推进政策扶持就业和鼓励促进就业。健全城乡公共就业服务体系，落实就业困难人员补贴政策，促进农村转移劳动力、城镇困难人员、化解产能过剩过程中职工安置就业。扶持小微企业，促进家庭服务业发展，发挥扩大就业积极作用。做好高校毕业生就业工作，就业意向签订率达到80％以上。二是推进社会保障体系建设。完善基本医疗、工伤、生育、失业保险市级统筹，健全医疗保险门诊统筹管理机制，将新农合和城镇居民医保财政补助标准由每人每年320元提高到380元。巩固城乡居民养老保险试点成果，提高群众自觉参保的积极性，引导激励适龄居民积极参保。做好被征地农民的社会保障工作。三是落实社会救助制度。建立社会救助工作部门联席会议、救助申请家庭经济信息比对制度，严格低保申请审核审批，推动设立市、县两级低收入家庭认定中心和救助信息多部门共享平台。适时提高城乡居民最低生活保障标准和补助水平，加大农村敬老院建设资金投入，提高五保集中供养率。四是发展各项社会事业。推进文明商丘建设，弘扬“商丘好人”精神，加强公民道德建设，践行社会主义核心价值观。实施文化惠民工程，开展文化下乡、文化进社区活动，加快县级数字影院建设，实现县（区）全覆盖。依托现有资源，完善提升市图书馆和青少年活动中心。完成商丘博物馆陈展并向社会公众开放，做好大运河（商丘段）世界文化遗产保护和展示工作。深化文化市场综合执法改革，持续开展“扫黄打非”，净化文化市场。优化城乡基础教育配置，调整城乡学校结构布局，新建改扩建初中13所、小学21所、九年一贯制学校2所，在商务中心区新建1所标准化幼儿园。推动高中教育、职业教育、民办教育加快发展，从秋季起免除全日制中等职业学校在校生的学费。促进高等教育协调发展，推动商丘医学高等专科学校专升本工作。做好重大传染病、慢性病防控救治和各类突发公共卫生事件应急处置，完善基层医疗卫生服务体系，让更多群众享受优质医疗服务。发挥住房公积金的保障作用，全市公积金归集覆盖率80％以上。坚持计划生育基本国策，推进免费孕前优生健康检查项目城乡全覆盖，提高出生人口素质，落实好单独二孩生育政策，促进人口长期均衡发展。稳定物价总水平，继续实行社会救助和保障标准与物价上涨相挂钩的联动机制。五是加强和创新社会治理。健全四项基层基础制度，推进县乡村三级服务体系建设。加强法治宣传教育和骨干队伍培训，完成“六五”普法任务。集中开展社会矛盾纠纷预防化解活动，健全分级管控、整体联动的预防化解体系。加强和改进信访工作，开展信访突出问题专项治理，解决人民群众的合理诉求，化解信访矛盾。完善社会治安防控体系建设，推进物防技防人防综合应用，完善“一村一警”长效机制，做好隐患排查治理和安全预防控制。做好反恐工作，依法严厉打击暴力犯罪、黑恶势力、“黄赌毒”等各类犯罪活动，确保社会和谐稳定、人民安居乐业。创新流动人口、特殊人群、网络虚拟社会服务和管理，引导社会组织有序参与社会治理，全面推进社区矫正工作。加强重大公共安全事件预警、应急、救援体系建设，不断提高预防和处置突发事件的能力。落实安全生产责任制，加强食品药品等行业监管，严格责任追究，确保不发生重特大安全事故。

　加强国防教育和国防后备力量建设，深入开展双拥共建，支持驻商解放军、武警部队、预备役部队、民兵和人民防空建设，促进军民融合深度发展。支持工会、共青团、妇联、工商联等人民团体发挥桥梁纽带作用。做好民族宗教、统计、外事侨务、对台、史志、地震、社会科学研究等工作。

　继续办好事关人民切身利益的“十件实事”：

　1．开工建设宋城路（紫荆路～归德路）、商都大道等30条新建道路，打通长江东路、胜利东路、香君西路等17条断头路，改造提升金桥路、归德南路（北海路～高速南出入口）等22条道路。

2．实施宋城路、长江西路等19条道路、132条背街小巷亮化工程。

3．开工建设汉梁文化公园、华夏游乐园，完成金世纪广场升级改造工程，完成包河和25条道路的景观带和廊道绿化。

　4．完成中心城区6条河道清淤扩容，实施蔡河、运河、中水渠贯通工程。完成第五污水处理厂二期工程，开工建设第六、第八污水处理厂。

　5．实施商丘火车站南广场、火车南站广场改造提升工程。

　6．新开工各类保障性住房32138套，其中公共租赁住房12061套，城市棚户区改造20077套。

　7．市区内建设水冲公厕50座。

　8．开工建设大剧院、群艺馆、科技馆、体育中心二期工程。

　9．购买公益性岗位5000个，城镇新增就业6万人，就业困难人员实现就业7300人。

　10．解决108万农村居民和学校师生的饮水安全问题。

四、加强政府自身建设

　按照“三严三实”要求，以建设法治政府、服务型政府为重点，推进政府自身改革建设，增强政府执行力和公信力。

　（一）转变职能，提高效率。进一步简政放权，继续清理行政审批事项，建立权力清单制度，清单之外的一律不得实施审批。强化政府在加强市场监管、公共服务、社会管理、环境保护等方面的职能，做到不越位、不缺位。推进诚信政府建设，大力弘扬“一言九鼎，实干兴商”城市精神，不断提高政府公信力，带动商业诚信和社会诚信。加强政府绩效管理，强化行政问效，坚决整治不作为、慢作为、乱作为，全面提升政府服务水平。

　（二）转变作风，务实重干。巩固党的群众路线教育实践活动成果，持续解决好“四风”问题。深化学习型机关建设，加大对新知识、新技术的学习培训，着力建设高素质公务员队伍。加强调查研究，善于把握和运用市场经济规律，抓住工作重点，调动各方力量，提高应对复杂局面的能力。大力倡导务实重干，坚持实事求是，真抓实干，不搞“形象工程”、“政绩工程”，努力创造经得起实践、人民和历史检验的实绩。

　（三）依法行政，科学决策。依法履行政府职能，敢于负责、勇于担当。坚决执行人大及其常委会决议决定，自觉接受法律和工作监督，支持政协参政议政，主动接受政协民主监督。认真办理人大代表建议和政协委员提案，广泛听取民主党派、工商联、无党派人士和人民团体意见。完善决策程序，重大事项由公众参与、专家咨询、风险评估、合法性审查和集体讨论决定，确保民主决策、科学决策。支持审计、监察部门依法独立行使监督权。深化政务公开，增强政府工作透明度。

　（四）廉洁从政，服务为民。坚持人民至上，强化服务意识和纪律意识，把守纪律、讲规矩摆在更加重要位置，严格执行中央“八项规定”和省市关于加强作风建设的各项规定。坚持从严从俭，厉行节约，反对浪费，严格控制“三公”经费。继续推行廉政监督员制度，强化对政府机关，尤其是对窗口单位、关键部门和重点岗位的监督。认真落实党风廉政建设责任制，强化“一岗双责”，把廉洁从政贯彻到政府工作各个环节。完善财政资金支出、工程项目招投标、土地规划管理等各项制度。坚决反对和惩治腐败，以反腐败斗争的实效保障发展、取信于民。

　各位代表！

　新的征程已经开启，新的目标催人奋进。让我们在省委、省政府和市委的坚强领导下，凝聚力量、开拓创新，埋头苦干、狠抓落实，推动商丘经济社会持续健康发展。
